# A Lineage of Begomoviruses Encode Rep and AC4 Proteins of Enigmatic Ancestry: Hints on the Evolution of Geminiviruses in the New World

**DOI:** 10.3390/v11070644

**Published:** 2019-07-13

**Authors:** Sandra Iliana Torres-Herrera, Angélica Romero-Osorio, Oscar Moreno-Valenzuela, Guillermo Pastor-Palacios, Yair Cardenas-Conejo, Jorge H. Ramírez-Prado, Lina Riego-Ruiz, Yereni Minero-García, Salvador Ambriz-Granados, Gerardo R. Argüello-Astorga

**Affiliations:** 1 División de Biología Molecular, Instituto Potosino de Investigación Científica y Tecnológica, A.C., Camino a la Presa de San José 2055, Lomas 4ta Secc, San Luis Potosi 78216, S.L.P., México; 2 Centro de Investigación Científica de Yucatán, A.C., Mérida 97000, Yucatán, México; 3 CONACYT–CIIDZA–Instituto Potosino de Investigación Científica y Tecnológica A.C., Camino a la Presa de San José 2055, Lomas 4ta Secc, San Luis Potosi 78216, S.L.P., México; 4 CONACyT-Universidad de Colima, Laboratorio de Agrobiotecnología, Carretera Los Limones-Loma de Juarez (s/n), Tecnoparque CLQ Colima 28629, Colima, México; 5 Facultad de Ciencias Forestales, Universidad Juárez del Estado de Durango, Río Papaloapan Esquina con Blvd Durango (s/n), Col. Valle del Sur. 34120, Durango, Dgo, México

**Keywords:** geminivirus evolution, replication protein, curtovirus taxonomy, endogenous viral element, replication origin, rolling-circle replication

## Abstract

The begomoviruses (BGVs) are plant pathogens that evolved in the Old World during the Cretaceous and arrived to the New World (NW) in the Cenozoic era. A subgroup of NW BGVs, the “*Squash leaf curl virus* (SLCV) lineage” (S-Lin), includes viruses with unique characteristics. To get clues on the evolutionary origin of this lineage, a search for divergent members was undertaken. Four novel BGVs were characterized, including one that is basal to the group. Comparative analyses led to discover a ~670 bp genome module that is nearly exclusive of this lineage, encompassing the replication origin, the *AC4* gene, and 480 bp of the *Rep* gene. A similar DNA module was found in two curtoviruses, hence suggesting that the S-Lin ancestor acquired its distinctive genomic segment by recombination with a curtovirus. This hypothesis was definitely disproved by an in-depth sequence analysis. The search for homologs of S-Lin Rep uncover the common origin of Rep proteins encoded by diverse *Geminiviridae* genera and viral “fossils” integrated at plant genomes. In contrast, no homolog of S-Lin Rep was found in public databases. Consequently, it was concluded that the SLCV clade ancestor evolved by a recombination event between a primitive NW BGV and a virus from a hitherto unknown lineage.

## 1. Introduction

Geminiviruses are plant viruses that possess small genomes composed by one or two circular molecules of single-stranded DNA [1] that are individually packed into geminate virions formed by two fused icosahedral hemicapsids [2,3]. The geminiviruses constitute the largest family of plant viruses, with over 450 distinct species that occur across all world regions with favourable climates for their insect vectors [4,5]. These viruses infect a multitude of wild plants in addition to a variety of agricultural species, causing significant reduction of food stocks and heavy economic losses in many countries [4,6]. The family *Geminiviridae* is divided into distinct genera based on the insect vector species, host range, genome organization, and phylogeny [7,8]. Nine genera are currently recognized by the International Committee on Taxonomy of Viruses (ICTV): *Becurtovirus*, *Begomovirus*, *Capulavirus*, *Curtovirus*, *Eragrovirus*, *Grablovirus*, *Mastrevirus*, *Topocuvirus*, and *Turncurtovirus* [5]. The begomoviruses (BGVs) represent the ecologically more successful subgroup, encompassing 409 recognized species [9]. BGVs infect dicotyledonous plants and are transmitted by the polyphagous vector *Bemisia tabaci*, a cosmopolitan complex of morphologically indistinguishable whitefly species [10,11] Two major phylogenetic groups of BGVs are generally recognized on the basis of geographical distribution and genome organization: the New World (NW: the Americas) and the Old World (OW: Africa, Eurasia, the Indian subcontinent, and Oceania) BGVs [12,13]. The NW BGVs (~140 species) possess a bipartite genome (DNA-A and DNA-B) with a few reported exceptions [14,15,16], whereas the OW BGVs include both bipartite and monopartite species, with predominance of the latter [12,17].

The genomic component A (or DNA-A) of NW BGVs displays five open reading frames (ORFs), one in the virion strand sense (*AV1* or *CP*) encoding the coat protein, and four in the complementary sense (*AC1* or *Rep*; *AC2* or *TrAP*; *AC3* or *REn;* and *AC4*) that encode proteins involved in a variety of functions including virus DNA replication, interference of plant cell cycle, temporal regulation of viral gene expression, and suppression of host antiviral responses [1,18]. The genomic component B (or DNA-B) contains only two genes: *BV1* or *NSP*, and *BC1* or *MP*, encoding proteins involved in the intracellular, intercellular, and systemic movement of the virus [19,20]. The genomic components of bipartite BGVs share a ~180 bp sequence known as “common region” (CR), which contains the plus-strand virus replication origin (*Ori*) composed by a collection of *cis*-acting sequences 5′-associated to a “stem-loop” element, where Rep cuts the plus-strand of viral DNA to initiate its replication by a rolling-circle (RCR) mechanism [21].

The NW BGVs display some features that clearly distinguish them from their Old World counterparts: (1) the absence of an *AV2* gene [13,22]; (2) a different number and arrangement of high-affinity Rep-binding sites (“iterons”) in the *Ori* region [23,24]; (3) a PWRsMaGT conserved motif in the CP N-terminal domain [22,25]; and (4) an AVRFATDK motif in the REn protein [26]. Notwithstanding those differences, diverse lines of evidence indicate that the BGVs native to the Americas evolved from OW ancestors [12,27]. In a comprehensive study that entailed both phylogenetic reconstructions and molecular clock-based methods of comparative genome analysis, Lefeuvre et al. [28] concluded that the NW BGVs evolved from Asian ancestors that probably arrived to the Americas in the Oligocene (30–35 MYA), when a land bridge between Asia and North America, and a climate favoring an unbroken belt of vegetation between those continents, presumably existed [28]. According to this scenario, the NW BGVs’ ancestors would have spread from Alaska into the two Americas, engendering several secondary BGVs lineages in the course of its evolutionary radiation. One of these clades, named after the *Squash leaf curl virus*, encompasses a number of BGV species, which are scattered from Southern USA to Argentina [29,30,31]. Members of the SLCV lineage (S-Lin, for simplicity) display two significant features distinguishing them from other NW BGVs: (a) the Rep-binding sites (iterons) are more numerous and display a unique arrangement in the *Ori* [23,32], and (b) the N-terminal domain of its Rep protein exhibits low aa sequence identity (<50%) with its putative homologous from other BGV lineages [24,30]. The evolutionary and functional significance of those distinctive characteristics has not been addressed up till now, notwithstanding its evident interest for a better understanding of BGVs’ evolution in the Americas. In this work, we describe the molecular characterization of four novel S-Lin viruses, which were systematically compared with all BGV sequences available at public databases. This comprehensive analysis allowed us to define with very high accuracy the distinctive features of the S-Lin members, and led to the intriguing conclusion that their common ancestor evolved by a recombination event between a virus from unknown ancestry and a primal NW begomovirus.

## 2. Materials and Methods

### 2.1. Sample Collection and Begomovirus Detection

Leaf samples of weeds with symptoms of virosis were collected during the summer of 2007 in several localities of the states of Yucatan and Morelos, as a part of a broad study to assess the diversity of BGVs in Southern México. Four field samples were selected for further analysis: (1) sample YP446, from a *Capraria biflora* (*Scrophulariaceae*) plant exhibiting yellow spots and mild leaf deformation, collected in Conkal, Yucatán (21°06′56″ N; 89°53′33″ W); (2) sample YP122, an *Abutilon permolle* (*Malvaceae*) plant showing mosaics in leaves, which was harvested in Suytunchen, Yucatán (18°81′17″ N; 98°98′33″ W); (3) sample YP534, a plant of *Jacquemontia pentantha* (*Convolvulaceae*) exhibiting mosaics and mild leaf distortion, collected in Mérida, Yucatán (21°01′42″ N; 89°38′1″ W); and (4) a *Vigna elegans* (*Fabaceae*) plant displaying yellow mosaics and stunting (sample Mor991) was collected in Yautepec, Morelos (18°50′17″ N; 98°98′59′03″ W). Total DNA was obtained from the dried samples using a modified Dellaporta protocol [33]. To increase the quantity of the field sample DNA, the extracts were subjected to rolling circle amplification (RCA) using the TempliPhi kit (GE Healthcare, Chicago, IL, USA). Polymerase chain reaction (PCR)-detection for begomoviruses was performed using Taq DNA Polymerase master mix (New England BioLabs, USA) and paired sets of lineage-specific primers, repSL2150for/cpYMAC-rev [30] and repDGR-for/cpYMAC-rev [34]. The PCR products were cloned in pGem-T Easy vector system (Promega, Madison, WI, USA). At least four clones obtained with the repSL2150for/cpYMAC-rev set of primers were sequenced for further analysis. A preliminary identification of viral sequences based on a BLAST search revealed that samples Mor991, YP122, YP446, and YP534 contained S-Lin BGVs significantly divergent in sequence from BGVs at NCBI databases. The full DNA-A sequence of S-Lin BGVs in the samples was obtained by PCR, using the pair of degenerate primers rep2370-rev/CP-EGP70-for, which produce amplicons overlapping the first PCR product along a ~600 bp long segment, thus encompassing the complete genomic component A. The DNA-B was PCR amplified using two sets of degenerate primers, BC1290for/BV1-MSKrev and BC1290-rev/BV1310-for (Table S1), which overlap in a 400 bp segment.

### 2.2. Cloning of Full-Length Viral Genomes

After the complete sequences of the DNA-A and DNA-B components of the S-Lin BGVs present in samples YP446, YP534, and Mor991 were assembled and verified, single restriction sites in each of the genomic components of those viruses were identified to design tail-to-tail (abutted) synthetic primers containing the selected endonuclease site at its 5′ end. The abutted primers are listed in Supplementary Table S1. The DNA extract of each field sample was subjected to amplification by PCR using the corresponding primers and Phusion^®^High-Fidelity DNA Polymerase (New England Biolabs, Ipswich, MA, USA) in the following conditions: 2 min at 94 °C; 35 cycles at 94, 56, and 68 °C for 30, 40, and 120 s, respectively; and a final extension of 5 min at 68 °C. The PCR products encompassing the complete viral genomic component were digested with the corresponding restriction endonuclease and subcloned into pBlueScript KS+, previously digested with the same endonuclease. After ligation and transformation in *Escherichia coli*, recombinant clones were verified by both double restriction pattern and sequencing.

### 2.3. Plant Infection Assays

*Nicotiana benthamiana* plants were inoculated using a low-pressure biolistic method. The target leaves (third to fourth leaf stage) were directly shot at either 100 to 120 psi helium pressure with tungsten particles (0.7 mm, BioRad, Hercules, CA, USA) covered with DNA-A and DNA-B of each virus (5 μg). Cloned monomeric components were treated with the corresponding endonuclease to release viral DNA prior to the biolistic inoculation. JacMYuV DNA-A was digested with *Bam*HI, DNA-B with *Hind*III; CarYSYV DNA-A with *Bst*I, DNA-B with *Sal*I; and ViMYV DNA-A with *Bam*HI, DNA-B with *Kpn*I. A total of eight seedlings were inoculated with each pair of homologous componentes. Mock inoculated negative controls were included for each experimental group. The inoculated plants were maintained in a growth chamber (27 °C, daily cycle of 16 h light/8 h dark) and scored for the appearance and development of disease symptoms during 3–5 weeks. All plants, both symptomless and symptomatic, were tested for the presence of viral DNA in new leaves at 14 dpi by PCR-based detection, using both DNA-A and DNA-B specific primers.

### 2.4. Phylogenetic Analyses

All evolutionary analyses were carried out using algorithms included in MEGA v10.0.4 [35]. The alignments of full-length DNA-A, Rep N-terminal domain (first 160 amino acid residues), and AC4 protein sequences were carried out using ClustalW algorithm with default parameters. The evolutionary history between the new viruses and related BGVs was inferred using either the neighbor-joining method [36] or the maximum likelihood method based on the Tamura–Nei model [37]. Phylogenetic analysis of Rep160 and AC4 proteins were conducted by using the maximum likelihood method based on the general reverse transcriptase model for Rep160 and the JTT matrix-based model for AC4. The substitution models were predicted by the best-fit substitution model (ML). In all cases, phylogeny tests were carried out by the bootstrap method (1000 replicates).

## 3. Results

### 3.1. Isolation and Characterization of Novel SLCV-Lineage (S-Lin) Begomoviruses

**Figure 1:**
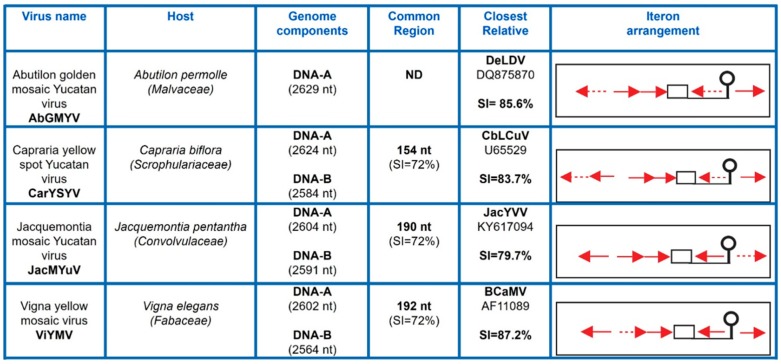
Summary of significant data on the begomoviruses described in this work. SI, sequence identity; ND, not determined (DNA-B is not known). Red arrows represent iterons (Rep-binding sites) and discontinuous arrows represent imperfect repeats; the TATA-box is represented by a square, and the conserved stem-loop element by a balloon-like figure.

Leaf samples of a variety of weeds exhibiting symptoms of virosis (e.g., leaf curling, yellowing, mosaics, interveinal chlorosis, growth stunting, and so on) were collected during the summer of 2007 in diverse localities of Yucatán and Morelos, México (See Section 2). The presence of BGVs was tested by PCR using a combination of both “universal” and “lineage-specific” primers designed to improve the detection of either S-Lin members or “typical” NW BGVs [30,34]. Nine out 16 examined samples produced amplicons of the expected size (~1.4 kb) with the pair of primers repSL-2150for/cpYMACrev, specific for detection of S-Lin members. Sequencing of those PCR products showed that four of them were significantly divergent (SI < 88%) from other BGV sequences available in public databases. Those distinct viruses were isolated from *Abutilon permolle* (sample YP122), *Capraria biflora* (sample YP446), *Jacquemontia pentantha* (sample YP534), and *Vigna elegans* (sample Mor991) plants, respectively (Figure S1). The complete DNA-A was obtained by sequencing a second amplicon (~1.9 Kb) generated with the pair of primers repSL2370rev/cpEGP70 for that overlapped the first amplicon along a ~600 bp segment. The complete sequence of the cognate DNA-B of three of those BGVs was obtained by a similar procedure (see Section 2). The assembled genomic sequences were compared with those from the NCBI database using BLASTn, and pairwise nucleotide sequence identities were calculated with SDTv1.2 [17]. The analyses showed that the closest relative of each of the four isolated BGVs exhibited a full length DNA-A sequence identity lower than 90% (Figure S2), hence indicating that these viruses are separate species, according to the current ICTV taxonomic criterion for begomoviruses [17]. The new BGVs were named *Abutilon golden mosaic Yucatan virus* (AbGMYV; GenBank accession number: KC430935), *Capraria yellow spot Yucatan virus* (CarYSYV; accession no. KC426927 and KC426928), *Jacquemontia mosaic Yucatan virus* (JacMYuV; accession no. JQ821386 and JQ821387), and *Vigna yellow mosaic virus* (ViYMV; accession no. KC430936 and KC430937), respectively. The four viruses exhibited the typical genome organization of New World BGVs, with a DNA-A containing one ORF (*CP*) on the virion-sense strand and four ORFs (*AC1*–*4*) on the complementary-sense strand; the DNA-B included two ORFs, *BV1* and *BC1*. In addition, all those BGVs exhibited the characteristic iteron arrangement of the SLCV clade [23,24], with two extra iterons flanking the stem-loop element at the *Ori* region (Figure 1). CarYSYV harbored six iterons with a TGGAGTCC consensus, whereas the five iterons of AbGMYV and JacMYuV displayed a TGGTGTCC sequence. In contrast, ViYMV exhibited five iterated elements with a TGGAGACC consensus, differing in the sixth nucleotide from the former BGVs iterons. In fact, ViYMV iterons are unique in sequence among the S-Lin members. The relevant information on the novel viruses is summarized in Figure 1.

### 3.2. Recombination Analysis

The DNA-A of the new BGVs was analyzed with the suite of programs for detection of recombinant breakpoints in the RDP3 package [38]. The analysis did not reveal significant evidence of recombination in CarYSYV, JacMYuV, and ViYMV. In contrast, one recombination event with breakpoints at positions 1969 and 2595 was identified in AbGMYV. One recombinant 626 nt long segment was presumably derived from *Desmodium leaf deformation virus* (DeLDV; the “minor progenitor”) encompassing the complete *AC4* ORF, the 5′ third of the Rep gene, and most of the common region (Figure S3). The remaining part of AbGMYV DNA-A was apparently derived from *Corchorus yellow spot virus* (CoYSV). It is noteworthy that both putative progenitors of AbGMYV are native to Yucatan Peninsula [39,40], and that CoYSV is not a member of the S-Lin. The recombination between CoYSV and DeLDV was well supported by six methods in the RDP3 package (GENECONV, *p* = 2.649 × 10^−34^; RDP, *p* = 3.606 × 10^−31^; BootScan, *P* = 6.429 × 10^−27^; MaxChi, *p* = 1.172 × 10^−27^; Chimaera, *p* = 1.636 × 10^−25^; 3Seq, *p* = 7.617 × 10^−50^).

### 3.3. Phylogenetic Relationships of the New Begomoviruses

**Figure 2:**
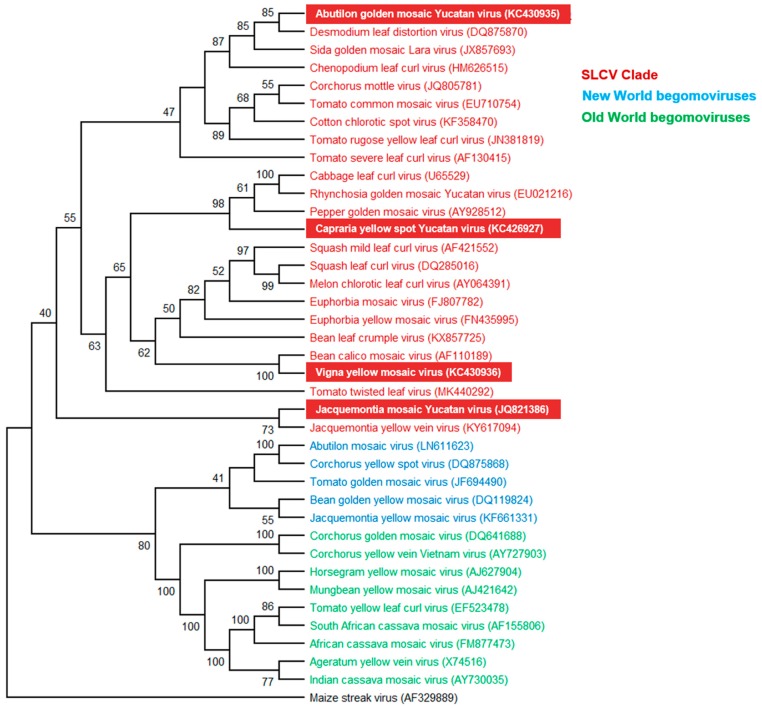
Phylogenetic relationships between the novel begomoviruses (highlighted in red background) and selected begomoviruses. Phylogenetic analysis was based on the alignment of the full-length DNA-A sequences of 24 members of the SLCV clade (highlighted in red), five begomoviruses (BGVs) from New World (highlighted in blue), nine BGVs from Old Word (highlighted in green), and one mastrevirus as outgroup (black). The phylogenetic tree was inferred using the neighbor-joining method. Bootstrap values (1000 iterations) are indicated for each node. GenBank accession numbers are indicated in parentheses.

A phylogenetic tree based on the DNA-A sequences of ~90 NW BGVs and 30 OW BGVs was generated using the neighbor-joining method with 1000 bootstraps replications. The resulting dendrogram [41] confirmed that the novel BGVs belong to the SLCV lineage. In Figure 2, a simplified phylogenetic tree including 24 S-Lin viruses and 14 “typical” (i.e., non S-Lin) NW and OW BGVs is illustrated.

CarYSYV grouped into a small cluster of Mesoamerican viruses that includes *Cabbage leaf curl virus* (CaLCuV) and *Pepper golden mosaic virus* (PepGMV); ViYMV was closely related to other legume-infecting BGVs, that is, *Bean calico mosaic virus* (BCaMV) and *Bean leaf crumple virus* (BLCrV); AbGMYV grouped into a subclade including both Mesoamerican and South American BGVs, while JacMYuV and *Jacquemontia yellow vein virus* (JacYVV) were basal to the clade. A phylogeny of the same DNA-A sequences that was reconstructed using the maximum likelihood method is illustrated in Figure S4. On the other hand, a phylogenetic tree based on comparisons of full-length DNA-B sequences was not utterly congruent with that reconstituted from the DNA-A. For instance, the DNA-B of JacMYuV clustered into the large AbMV clade of NW BGVs, whereas CarYSYV was closer to the PHYVV cluster (Figure S5). Incongruent phylogenies of DNA-A and DNA-B components of a bipartite begomovirus are generally explained by recombination and/or re-assortment events along the evolutionary history of that particular species [12].

### 3.4. Infectivity of Cloned DNA-A and DNA-B Components

**Figure 3:**
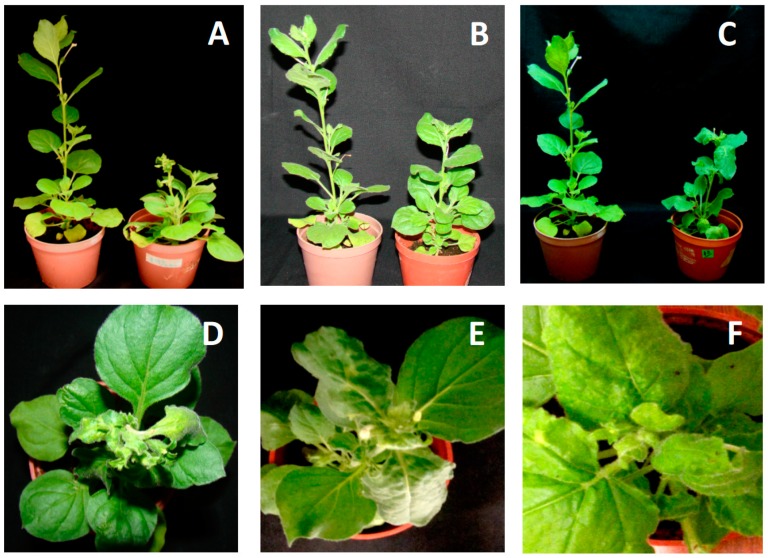
Nicotiana benthamiana plants inoculated with DNA-A and DNA-B of (**A**,**D**) JacMYuV, (**B**,**E**) CarYMYV, and (**C**,**F**) ViMYV. The symptomatic plants (28 dpi) (right side of photography) are compared with mock-inoculated plants (left side).

Full-length clones of both genomic components of JacMYuV, CarYSYV, and ViYMV were generated to verify their capability to produce infections in plants. The infectious clones were obtained via PCR amplification using back-to-back primers designed on unique restriction sites (see Section 2). Biolistic inoculation of *Nicotiana benthamiana* plants with clones of JacMYuV DNA-A and DNA-B produced clear symptoms at 10 dpi in all tested plants (8/8). Disease signs included strong shortening of internodes, and yellowish and severe deformation of new leaves (Figure 3A). Symptoms did not diminish with time, and plants did not developed flowers even after 40 dpi. In contrast, six out of eight plants bombarded with CarYSYV clones developed mild symptoms, including a slight delay in growth and mild leaf curling (Figure 3B), which lessened gradually after 20 dpi, suggesting remission. Finally, the clones of ViYMV produced symptoms in six out of eight inoculated plants, which exhibited mild chlorosis and deformation of new leaves at 12 dpi, although remission of symptoms was noticed after 25 dpi (Figure 3C). These results showed that the three tested BGVs have different capabilities to induce symptoms in the common host *N. benthamiana*, and confirmed the complementary nature of the isolated viral genomic components. This was an important issue in the case of CarYSYV, whose common region (CR) exhibited a low conservation in sequence (SI = 72%) between cognate genomic components, as previously indicated (see Figure 1).

### 3.5. Identification of a Distinctive Rep Domain Encoded by SLCV-Lin BGVs

**Figure 4:**
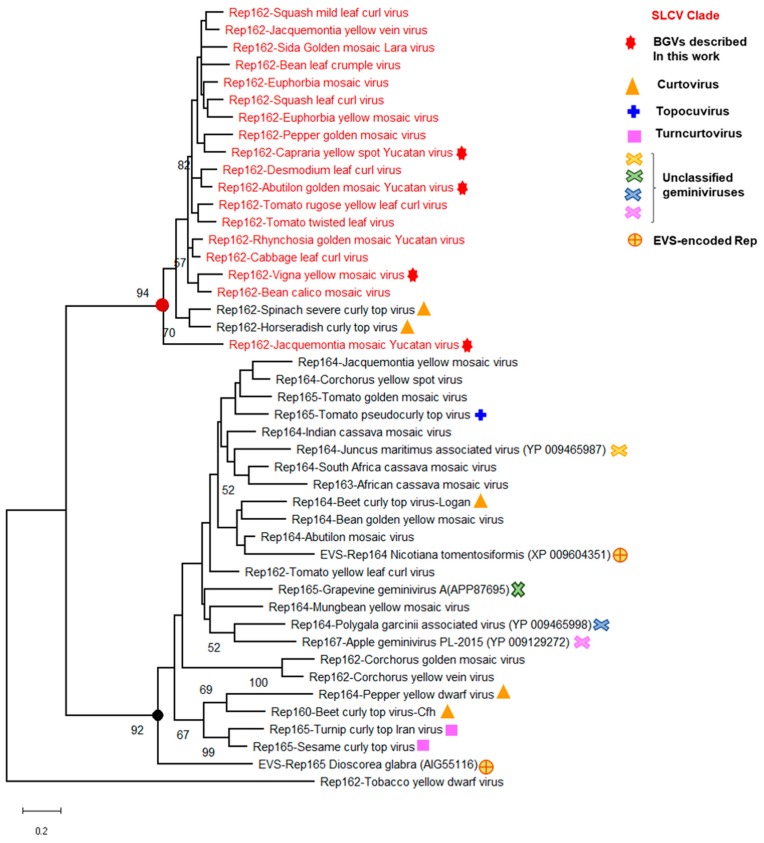
Phylogenetic relationships between the Rep N-terminal domain (aa 1–162) of SLCV-Lin members (highlighted in red) and its homologous of other geminivirus subgroups. The phylogenetic tree was inferred using the maximum likelihood method based on the general reverse transcriptase model. The percentage of trees in which the associated taxa are clustered together is shown next to the branches (1000 iterations). The red filled circle indicates the node of Rep162 of the SLCV cluster, and the black filled circle indicates the main branch of equivalent Rep162 domains of other geminiviruses. EVS, endogenous viral sequences.

To define with higher accuracy the genomic and molecular hallmarks of the SLCV lineage members, the intergenic region and the predicted proteins encoded in the DNA-A of all its members were systematically compared with those from other NW BGVs. This broad analysis revealed a number of S-Lin specific motifs in the Rep N-terminal domain (aa 1–160), but not in other domains of that protein. The expansion of the comparative analysis to include Rep proteins encoded by OW BGVs from distinct lineages, such as the so-called Corchoviruses [22,25], Legumoviruses [42], Sweepoviruses [12,17], and other BGVs indigenous to OW landmasses, confirmed that the identified S-Lin Rep motifs are actually unique among the replication proteins encoded by BGVs [30]. Consequently, the comparative analysis was extended to embrace the Rep proteins of all *Geminiviridae* genera, looking for a plausible ancestor of the S-Lin Rep. A cladogram derived from the comparisons of Rep N-domain (aa 1–160) encoded by representative members from different geminivirus genera is illustrated in Figure 4. Several data of this phylogeny are noteworthy: (1) the Rep160 of SLCV-Lin viruses form a cluster clearly separated from the main branch of the equivalent Rep domain of geminiviruses belonging to four genera: *Begomovirus, Curtovirus, Topocuvirus*, and *Turncurtovirus*; (2) The Rep160 of JacMYuV is basal to the SLCV cluster; (3) the Rep160 of two curtoviruses, *Horseradish curly top virus* (HrCTV) [43] and *Spinach severe curly top virus* (SpSCTV) [44], are clustered within the S-Lin Rep branch. This observation naturally suggests that the distinctive Rep domain of S-Lin BGVs could be derived from a curtovirus related to HrCTV, a presumption that was later examined at close detail (see below).

### 3.6. Comparative Analysis of the AC4 Protein of SLCV Lineage Viruses

**Figure 5:**
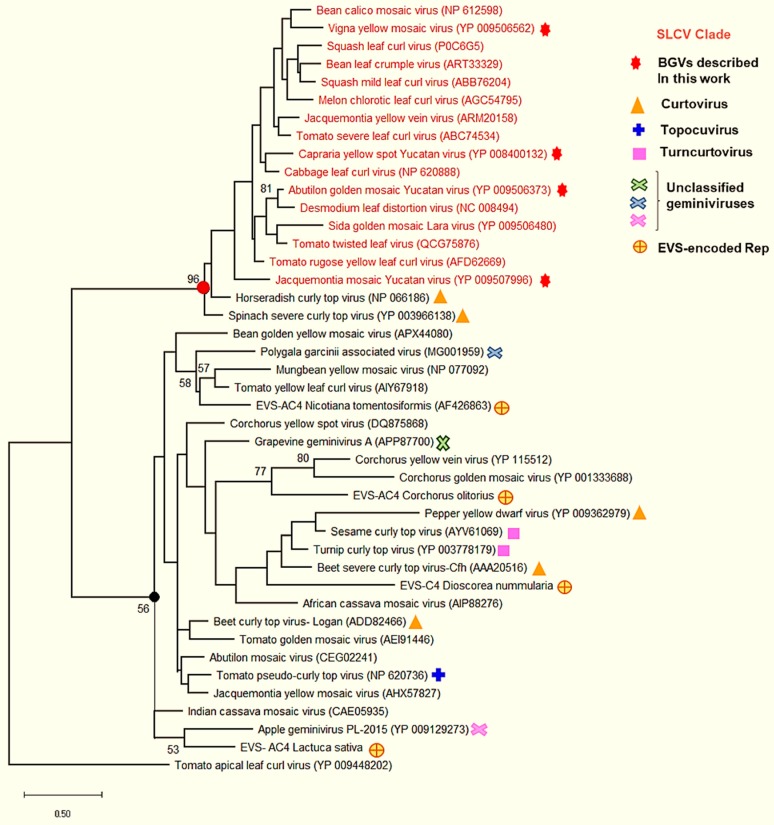
Phylogenetic relationships between AC4 proteins encoded by SLCV-Lin members (highlighted in red) and AC4/C4 proteins of other geminivirus subgroups. The phylogenetic tree was inferred by using the Maximum Likelihood method based on the JTT matrix-based model. The percentage of trees in which the associated taxa clustered together is shown next to the branches (1000 iterations). Red filled circle indicates the node of the S-Lin BGVs, and the black filled circle indicates the main branch of other geminiviruses.

As the *AC4* gene is entirely included in the DNA segment encoding the Rep N-domain, it may be anticipated that AC4 proteins of S-Lin BGVs should be divergent from other geminivirus AC4/C4 proteins. In fact, the alignment of the S-Lin AC4 proteins with their putative equivalents in other geminiviruses revealed a level of amino acid sequence identity so low (<25%) that they would hardly be considered homologous (i.e., evolutionarily related). In the cladogram illustrated in Figure 5, which was derived from comparisons of AC4/C4 proteins encoded by members from different *Geminiviridae* genera, the S-Lin AC4 proteins form a cluster clearly separated from other geminivirus AC4/C4 proteins. The hypothesis that S-Lin AC4 proteins are not evolutionary related to other AC4/C4 proteins was strongly supported by two distinct observations: (1) the former proteins are significantly larger (~120 aa) than AC4/C4 proteins encoded by “typical” BGVs, topocuviruses, turncurtoviruses, and some curtoviruses, which are 85 aa long on average; (2) S-Lin AC4 proteins lack the *N*-myristoylation motif (MGXLIS) that is critical for geminivirus AC4/C4 function as suppressor of silencing and determinant of pathogenesis [45,46,47]. Therefore, it is plausible that S-Lin AC4 proteins are neither homologous nor analogous (i.e., with similar functions) to other geminivirus AC4/C4 proteins.

### 3.7. Comparative Analysis of Curtovirus and S-Lin BGV Genomes

The striking similarity of the N-domain of Rep proteins encoded by S-Lin BGVs and the curtoviruses HrCTV and SpSCTV could be explained by assuming that the former BGVs evolved by a recombination event between a curtovirus and a begomovirus. As the full Rep proteins of SpSCTV and HrCTV display a sequence identity (i.e., 80%) that is practically equal to that showed with the Rep encoded by some S-Lin BGVs (e.g., *Tomato rugose yellow leaf curl virus*; FN434438), we inferred that the hypothetical recombination event plausibly encompassed the entire Rep gene, including the part of *C2* ORF that overlaps with the latter gene. Consequently, we search for any evidence of an evolutionary relationship between HrCTV and SpSCTV *C2* ORF and *AC2/C2* genes of begomoviruses. BLASTp analysis of HrCTV *C2* did not produce any significant data. In contrast, computer-assisted analysis of SpSCTV C2 revealed that residues in the protein N-terminus are conserved in AC2/C2 proteins of several Old World BGVs. For instance, the segment (aa 1–12) of SpSCTV C2 protein (i.e., MPYSFPSVNHCT) was 75% identical to the equivalent domain of the AC2 protein of one isolate of *East African cassava mosaic virus* (GenBank accession no. CBA13492), namely, MPsSsPSkNHCT. Moreover, homologous proteins encoded by several BGVS native to Asia (e.g., AYVV and MYMIV), the Mediterranean basin (e.g., TYLCV), and Australia (e.g., ToLCV) display a consensus sequence, MxxSxPSxNHCT, at their N-terminus. The real homology of SpSCTV *C2* and begomovirus *AC2/C2* genes was confirmed by in silico translation (in different frames) of the DNA segment where *C1* and *C2* ORFs overlap. Indeed, the translation of the 5′ end of SpSCTV C2 ORF in the frame of *C1* ORF produced the sequence AVFISISEPLY, which is 82% identical to the Rep segment 330–340 of *Sri Lankan cassava mosaic virus* (AtFISlSEPLY), 332–342 of *Jatropha mosaic Nigeria virus *(AtTFItISEPLY), 331–341 of *Tomato leaf curl China virus* (AVFvSItEPLY), and 335–345 of *Asystasia mosaic Madagascar virus* (AtFItISEPLY), among others. Taken together, the results from this systematic sequence analysis did not provide support for the notion that S-Lin BGVs acquired their distinctive genomic module from a curtovirus; on the contrary, the analysis data strongly support the hypothesis that HrCTV and SpSCTV got its atypical *Rep* and *C4* genes through recombination with an S-Lin begomovirus.

### 3.8. Searching for the Common Ancestor of the SLCV Clade Members

**Figure 6:**
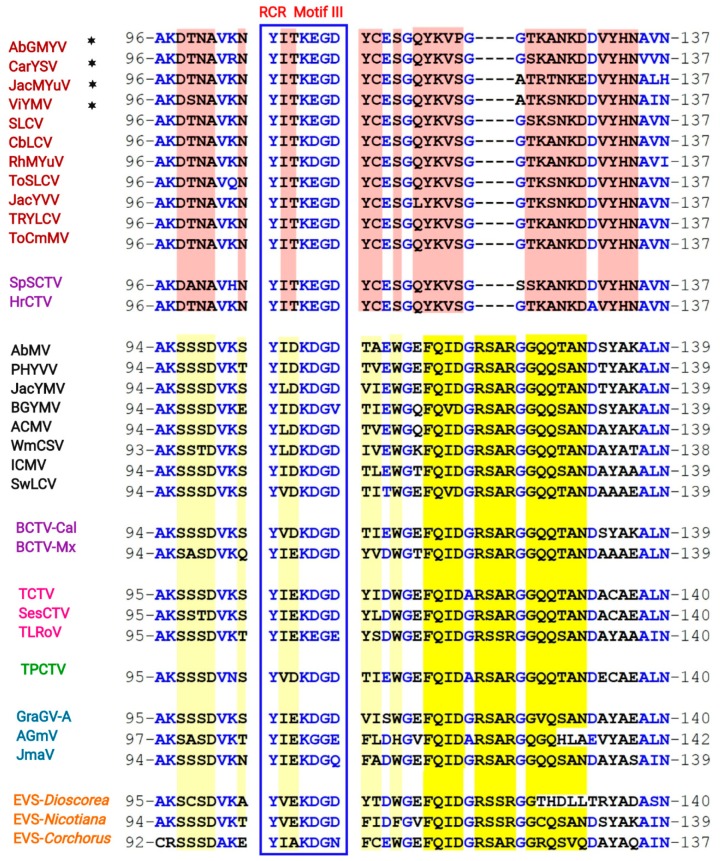
Alignment of the RCR motif III-containing region of Rep proteins encoded by S-Lin BGVs; other NW and OW begomoviruses; members of the genera *Curtovirus, Turncurtovirus*, and *Topocuvirus*; three unclassified geminiviruses; and several endogenous viral sequences (EVS). The distinctive signature of 17–18 aa residues distinguishing two major evolutionary lineages of geminiviruses is highligted in light magenta and yellow color, respectively. The name and GenBank accession numbers of the geminiviruses are listed in Supplementary Table S2.

Having ruled out the possibility that a curtovirus related to SpSCTV was the evolutionary ancestor that transferred the atypical genomic module to S-Lin BGVs, we proceeded to search for endogenous viral sequences (EVS) in higher plant genomes, hoping to find some molecular “fossil” with significant similarity with S-Lin BGV sequences. Geminivirus-related EVS have been described in *Nicotiana tomentosiformis* and its relatives [48,49], and in several species of the monocot *Dioscorea* sp. [50]. In addition, the survey of plant genome databases with a variety of bioinformatics tools allowed us to find out Rep-related proteins encoded in the genomes of *Lactuca sativa*, *Corchorus olitorious*, *Coffea canephora*, and *C. arabica.* A close examination of those molecular fossils did not reveal sequence motifs similar to those of S-Lin Rep; in actual fact, several of those EVS-encoded Reps exhibit motifs alike those of “typical” begomoviruses (Figure 6). A systematic analysis of multiple sequence alignments, including hundreds of predicted Rep proteins, disclosed the existence of two major groups of geminivirus Reps, based on the presence of one specific conserved sequence in the 116–134 domain. The distinctive signature of proteins encoded by members of four genera and three unclassified geminiviruses was the conserved consensus FQIDGRSARGGQQ(S/T)AND, of indeterminate function. Its counterpart in Rep proteins, encoded by S-Lin BGVs and the curtoviruses HrCTV and SpSCTV, was a 18 aa long motif, QYKVSGGTKANKDDVYHN (Figure 6). Those two distinctive aa sequences are close to the C-end of the conserved RCR Motif III, which contains the endonuclease active site of geminivirus Rep and a plethora of virus-encoded initiators of rolling-circle replication [51,52].

**Figure 7:**
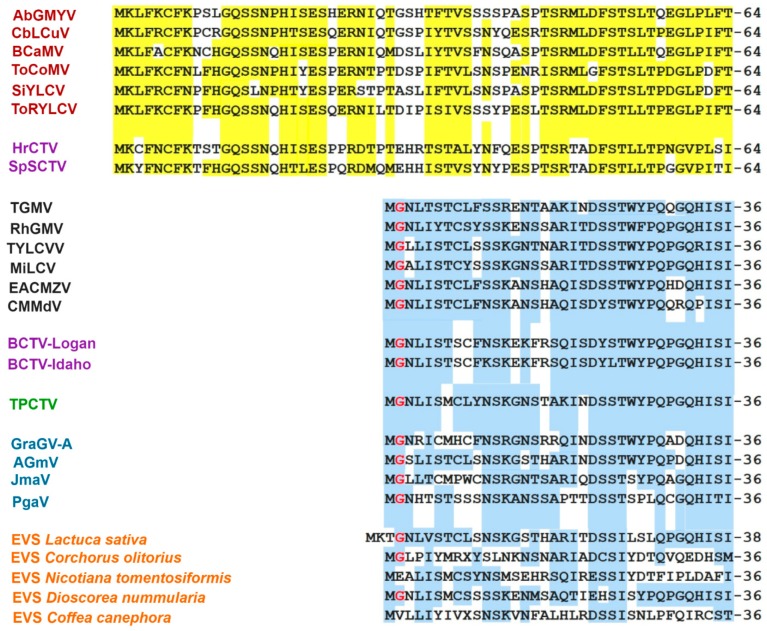
Alignment of the N-terminal domain of AC4/C4 proteins encoded by members of the S-Lin; other NW and OW begomoviruses; members of the genera *Curtovirus*, *Turncurtoviru*s, and *Topocuvirus*; four unclassified geminiviruses; and a number of endogenous viral sequences (EVS). The amino acid residues identical or similar are shadowed in yellow or blue to highlight the resemblances between the aligned protein sequences. The glycine residue of the N-myristoylation motif is marked in red. The name and GenBank accession numbers of the geminiviruses and EVS are enumerated in Supplementary Table S3.

In view of the fact that the Rep gene segment encoding the protein N-domain overlaps with the *AC4* gene, we look for significant correlations between the distinctive Rep 116–134 domain and the type of AC4/C4 protein. In all examined cases, viruses encoding Rep proteins with a QYKVSGGTKANKDDVYHN consensus also encode large AC4/C4 proteins lacking a N-myristoylation motif, whereas viruses having Rep with a FQIDGRSARGGQQ(S/T)AND sequence usually encode AC4/C4 proteins with a N-myristoylation motif (Figure 7). This rule holds even in the case of Rep and AC4/C4 proteins encoded by EVS integrated in plant genomes, as we were able to know by reconstructing the encoded proteins from the EVS nucleotide sequences (Figure 6 and Figure 7).

## 4. Discussion

It is generally accepted that the begomoviruses evolved in the Cretaceous period, prior to the tectonic breakup of the supercontinent Gondwana into the landmasses of India, Africa, South America, Antarctica, and Australia [13,19,27]. This supposition is based on the observation that phylogenies generated from full-length DNA-A alignments consistently produce continent-specific lineages [12,13]. The NW BGVs form a separate major branch, with several distinct clades that are not restricted to specific geographical areas of the Americas. The prevalent view on the origin of NW BGVs is that they evolved from Asian ancestors that arrived to North America probably ~30 MYA, according to the most comprehensive study on this subject that has been performed at present [28]. However, an alternative scenario, that is, that the BGVs arrived to the New World by South America when land connections with a warm Antarctic continent still existed, is likewise supported by several molecular, biogeographical, and paleovirological lines of evidence [53]. Regardless of which of the two alternative scenarios may be true, the evolutionary radiation of BGVs took place during its spread across the two Americas, and eventually some secondary lineages emerged, such as the so-called clades of the SLCV, AbMV, and PHYVV; as well as others identified in diverse phylogenetic studies [27,54]. In this work, we have established, through the genome characterization of four novel BGV species and comprehensive analyses based on a variety of comparative approaches, that the SLCV clade members are quite different from other NW and OW BGVs in a number of molecular traits, including the replication origin region, and the genes encoding the AC4 protein and the Rep N-domain. Because all those elements are comprised in a compact, unbroken genome module ~670 nt long, it is feasible to analyze it as an evolutionary unit. Thus, the significant divergence of the DNA-binding domain of the S-Lin Rep [24] correlates with the peculiar characteristics of their iterons, which differ from those of other BGVs in two aspects: (a) its number and arrangement within the *Or*i region; (b) its nucleotide sequence, which exhibits a highly conserved consensus, TGGWG(T/a)CC, clearly different to the highly variable aatyGGNRNN iteron consensus of typical NW and OW BGVs [23,24,30]. Likewise, the atypical AC4 protein encoded by the S-Lin BGVs, which lacks a *N*-myristoylation motif, is inseparable from the peculiar Rep N-domain because the corresponding ORFs are overlapped.

In addition to its bizarre iterons, the CR of S-Lin members generally lacks the G-box adjacent to the *Ori* stem-loop element present in most NW BGVs, which is a critical activating element of TGMV *Rep* promoter [55]. Instead, most S-Lin BGVs exhibit a conserved GGGGCAAAA motif contiguous to the *Ori* stem-loop [30]. To ascertain the probable function of this conserved (G4CA4) box, a search in databases specialized in plant *cis*-regulatory elements was carry out; in two of those databases, SoftBerry [56] and PLACE [57], the sequence GGGGCAAAA was identified as a binding-site for the transcription factor E2F of *N. tabacum* [58] and *Arabidopsis thaliana* [59]. Importantly, E2F transcription factors are key components of the molecular machinery responsible for plant cell cycle progression [58,59]. Furthermore, the position of the putative E2F binding site in the common region of S-Lin BGVs is consistent with the general occurrence of *cis*-regulatory elements in the immediate vicinity of the conserved “hairpin” sequence of geminivirus replication origin [23,32,55,60].

### Evolutionary Relationships of Curtoviruses with S-Lin Begomoviruses

The genus *Curtovirus* comprises four recognized species: BCTV, PepYDV, HrCTV, and SpSCTV; all of them native to USA and México [5,61,62] The extraordinary resemblance of Rep and AC4 proteins of SLCV-Lin BGVs with the homologous proteins of HrCTV and SpSCTV led to supposing that those BGVs evolved by recombination with a curtovirus. Nevertheless, this hypothesis does not explain several incongruous facts: (1) the Rep proteins of HrCTV and SpSCTV are very divergent from those of BCTV and PepYDV, displaying a sequence identity lower than 45% along its N-terminal (1–160) domain; (2) the *C4* gene of HrCTV and SpSCTV encodes a protein similar to that of S-Lin BGVs, lacking the N-myristoylation motif present in the C4 proteins of BCTV and PepYDV; (3) the *C2* ORF of HrCTV and SpSCTV encodes a ~135 aa protein with no apparent homology (i.e., less than 15% of aa sequence identity) with C2 proteins of BCTV and PepYDV. Moreover, even though SpSCTV and HrCTV have a *C1* ORF encoding highly similar Rep proteins (SI = 80%), their predicted C2 proteins display very low sequence identity (26%). In this work, it was demonstrated that HrCTV and SpSCT are actually recombinants of a curtovirus with a JacMYuV-like begomovirus, according to the phylogenetic relationships of their C4 and Rep160 proteins depicted in Figure 4 and Figure S6. The gathered evidence supporting this hypothesis is convincing because the *C1*/*Rep* genes of HrCTV and SpSCTV are definitely homologous to S-Lin BGV *Rep* gene, and the SpSCTV DNA region where the *C1* and *C2* ORFs overlap could be translated in silico to a short aa sequence highly similar to the N-terminus of AC2 proteins encoded by several BGVs, and to the 330–340 domain of Rep proteins encoded by the overlapping *C1* gene. Two important corollaries of the notion that HrCTV and SpSCTV derived from a common, recombinant ancestor, are the following: (1) the genus *Curtovirus* is not a monophyletic taxon, and (2) the *C2* ORFs of SpSCTV and HrCTV have undergone an evolutionary divergence that is by far higher than that in the overlapped *C1* ORF, which was jointly acquired by recombination. Both conclusions are important and should be further examined.

On the other hand, the curtoviruses BCTV and PepYDV possess *C2* and *C3* genes that are clearly homologous to those of begomoviruses. The notion that the *C2–C3* genes were acquired by those curtoviruses through recombination with a begomovirus has been broadly documented [13,62]. Considering the native geographic distribution of the curtovirus and its vector insect, the leafhopper *Circulifer tenellus*, it is natural to suppose that the intermolecular recombination events that independently gave rise to BCTV-PepYDV and HrCTV-SpSCTV were possible once the populations of the insect vectors, *Bemisia tabaci* and *Circulifer tenellus*, converged in the same geographical area (i.e., North America). Begomoviruses probably existed in South America before the formation of Panama Isthmus (~3 MYA) because the genomes of *Nicotiana tabacum* and its South American relatives contain endogenous begomoviral sequences that presumably integrated at plant genomes 3–6 MYA [28,49]. Consequently, it is reasonable to assume that modern curtoviruses evolved after the above mentioned geological event, which enabled plant and animal species to migrate between the two Americas by creating an intercontinental land bridge. Similarly, *Tomato pseudocurly top virus*, native to Florida and the only member of the genus *Topocuvirus*, has *C1–4* genes of apparent begomoviral origin [63], and probably evolved after the formation of Panama Isthmus.

**Figure 8:**
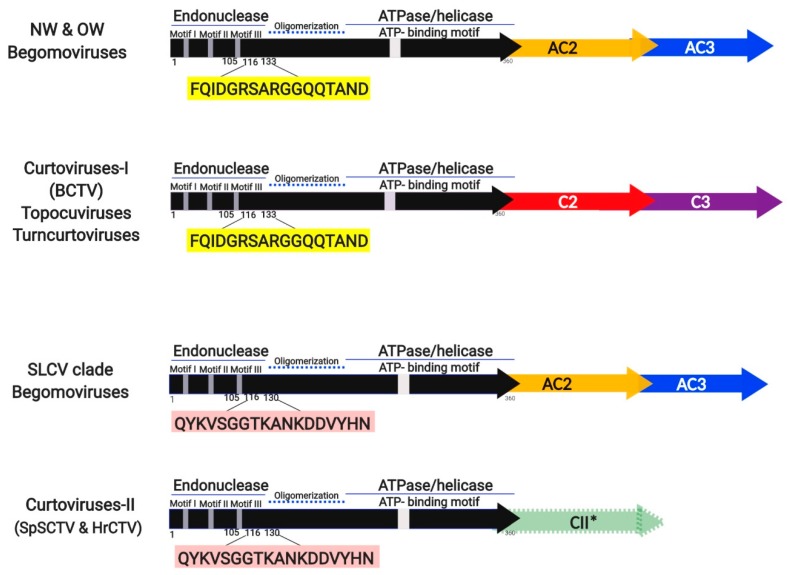
Simplified representation of complementary sense genes of begomoviruses and other subgroups of geminiviruses. The characteristic amino acid sequence signature in the aa112–130 domain of Rep proteins uncovered in this study is highlighted in color boxes. *AC2* and *AC3* genes of begomoviruses are represented by arrows in yellow and blue color, respectively. The *C2* and *C3* genes of members of other *Geminiviridae* genera are differently colored to emphasize structural and/or functional divergence from its begomoviral homologous. The green arrow of SpSCTV and HrCTV curtoviruses represents the highly divergent *C2* (i.e., CII*) open reading frame (ORF), of unknown function. NW, New World; OW, Old World.

The data derived of the extensive comparisons of Rep and AC4/C4 proteins performed in this study provide a useful background to think about the complex evolutionary history of the geminiviruses. In Figure 8, a simplified, schematic representation of some our findings is illustrated.

## 5. Conclusions

The members of the SLCV clade (35 species) encode unique AC4 and Rep proteins whose evolutionary origin is enigmatic. The actual function of that atypical AC4 protein is unknown, but probably differs from those of other AC4/C4 proteins of begomoviruses and curtoviruses. The replication origin region of the S-Lin BGVs harbors peculiar iterons and a putative binding site for E2F transcription factors, involved in the plant cell cycle, which is adjacent to the conserved stem-loop sequence. A virus basal to the SLCV lineage, JacMYuV, which is the closest relative to the hypothetical begomoviral ancestor of two recombinant curtoviruses, was characterized in this work. With the exception of the S-Lin BGVs, most geminiviruses displaying a Rep gene without introns encode proteins with a FQIDGRSARGGQQ(**S/T**)AND related consensus near the C-end of the endonuclease active site (i.e., the RCR Motif III). The actual function of the former 17aa long motif sited betwen the endonuclease and the oligomerization domains of Rep is unclear, although a study of TGMV Rep protein showed that specific mutations in the so-called “conserved sequence” (i.e., FQVDGRSARGGCQT) impaired several key Rep activities, including DNA-binding and cleavage, and protein oligomerization [64]. These observations suggest that the Motif III-associated conserved sequence is a critical subdomain of begomovirus replication proteins.

## Supplementary Materials

The following are available online at https://www.mdpi.com/1999-4915/11/7/644/s1, Figure S1: Weeds infected with the begomoviruses described in this study, Figure S2: Phylogenetic tree and matrix of pairwise sequence identity based on DNA-A sequences of the isolated begomoviruses and their relatives, Figure S3: Recombination event in the AbGMYuV DNA-A detected by RDP, Figure S4: Phylogenetic relationships between the novel begomoviruses and selected New World and Old World begomoviruses, Figure S5: DNA-B inferred evolutionary relationships of viruses isolated in this work compared with selected begomoviruses. Figure S6: Phylogenetic relationships between AC4 proteins encoded by SLCV-Lin members (highlighted in red) and those of other geminivirus subgroups, Table S1: Oligonucleotides used in this work, Table S2: Names, acronyms and GenBank accession numbers of geminiviruses and endogenous viral sequences (EVS) included in the alignments of Rep motif III region and the N-terminal end of AC4 proteins (Figure 6), Table S3: Names, acronyms and GenBank accession numbers of geminiviruses and endogenous viral sequences (EVS) included in the alignments of the N-terminal domain of AC4/C4 proteins (Figure 7).
