# Supplementary material for: A Lineage of Begomoviruses Encode Rep and AC4 Proteins of Enigmatic Ancestry: Hints on the Evolution of Geminiviruses in the New World
Source: Viruses. 2019 Jul 13;11(7):644. doi: 10.3390/v11070644 (PMC6669703; doi:10.3390/v11070644)
Supplement: Supplementary file 1 [file viruses-11-00644-s001.zip › Suppl. Table S1- Torres-Herrera et al..pdf]

A lineage of begomoviruses encode Rep and AC4 proteins of enigmatic ancestry: hints on the evolution of geminiviruses in the New World.

Iliana Torres-Herrera[1*], Angélica Romero-Osorio[1*], Oscar Moreno-Valenzuela[2], Guillermo Pastor Palacios[3], Yair Cardenas-Conejo[4], Jorge H. Ramírez-Prado[2], Lina Riego-Ruiz[1], Salvador Ambriz-Granados[1], Gerardo R. Argüello-Astorga[1&].

Supplementary Table 1. Oligonucleotides used in this work.

Table S1

Oligonucleotide	Sequence 5'- 3'
CP-YMAC-Rev	TTWGASGCATGNGTACATGCCA
CP-EGP70-For	GGTTGTGAAGGNCCNTGTAAGGTYCA
Rep-SL2150-For	GACGGCRTTGGYGTCTTTGGCWGC
Rep-SL2370-Rev	CTGYCRTGGTCAGTCGTCAAACC
BC1- 290-For	GAGAARTAGTGGAGATCTATGTTRCAYCT
BVI-310-Rev	CAATYTTRACNGTACCYTTRAAACG
BV1-310 For	TTYAAGGGTACCGTTAAGATTG
BC1-290 Rev	CCSATMAGRTGYAACATAGATCTCC
BC1-DLH- For	GGAAGCTGARAARTAGTGGAGATCTATG
BC1- PWK-For	TCGCAAACTCKGTAATAGAGTTTCCATGG
BV1-GATP- Rev	TTRGGGGTAGMACCGTCMATGTTCATATC
BV1- MSKA-Rev	CGCCTTCGACATMGTRTCWGACATCCAAC
JacA-Bam HI-For	ACT**GGATCC**CCTGCCTTAATGATTGC
JacA- Bam HI Rev	TTA**GGATCC**AGTAAGATGGATAACC
JacB–Hind I–For	TAT**AAGCTT**TCTCTCCCATCTCCCGT
JacB– Hind I Rev	AGA**AAGCTT**ATCAGATGCGCATCCATG
CapA-Bst XI-For	TAC**CCA**TCGTGT**TGG**TAAGCGTTTCTGC
CapA- Bst XI- Rev	TTA**CCA**ACACGA**TGG**GTAATGCCATTGC
CapB Sal I-For	ACT**GTCGA**CAGCTTTAACTTTCCTTTG
CapB-Sal I-Rev	TCT**GTCGAC**AGCTAAGCACTCTGTAG
VignaA-BamHI-For	AT**GGATCC**TCTATCCTGTATATC
VignaA BamHI-Rev	AGA**GGATCC**AATATACACAAGGA
VignaB-KpnI-For	TA**GGTACC**GTCAAGATTGAGCGTGTAC
VignaB-KpnI-Rev	TT**GGTACC**CTTGAAACGGAGTCG
