# Supplementary material for: A Lineage of Begomoviruses Encode Rep and AC4 Proteins of Enigmatic Ancestry: Hints on the Evolution of Geminiviruses in the New World
Source: Viruses. 2019 Jul 13;11(7):644. doi: 10.3390/v11070644 (PMC6669703; doi:10.3390/v11070644)
Supplement: Supplementary file 1 [file viruses-11-00644-s001.zip › Suppl. Table S2- Torres Herrera et al..pdf]

A lineage of begomoviruses encode Rep and AC4 proteins of enigmatic ancestry: hints on the evolution of geminiviruses in the New World.

Iliana Torres-Herrera[1,5*], Angélica Romero-Osorio[1*], Oscar Moreno-Valenzuela[2], Guillermo Pastor Palacios[3], Yair Cardenas-Conejo[4], Jorge H. Ramírez-Prado[2], Lina Riego-Ruiz[1], Yereni Minero-García[2], Salvador Ambriz-Granados[1], Gerardo R. Argüello-Astorga [1&].

[1] División de Biología Molecular, Instituto Potosino de Investigación Científica y Tecnológica, A.C., San Luís Potosí, SLP, México.

[2] Centro de Investigación Científica de Yucatán, A.C., Mérida, Yucatán, México

[3] CONACYT–CIIDZA–Instituto Potosino de Investigación Científica y Tecnológica A.C., San Luis Potosí, SLP, México,

[4] CONACyT-Universidad de Colima, Colima, México.

[5] Facultad de Ciencias Forestales, Universidad Juárez del Estado de Durango, México.

Supplementary Table S2. Names, acronyms and GenBank accession numbers of geminiviruses and endogenous viral *sequences (EVS)* included in the alignments of Rep motif III region and the N-terminal end of AC4 proteins (Figure 6)

Table S2 – Geminiviruses and endogenous viral sequences harboring a characteristic consensus in the Rep domain adjacent to Motif III

Virus	Acronym	GenBank Accession
**SLCV clade begomoviruses** **QYKVSGGTKANKDDVYHN**		
Virus	Acronym	GenBank Accession
Abutilon golden mosaic Yucatan virus	AbGMYV	KC430935
Capraria yellow spot Yucatan virus	CarYSYV	KC426927
Jacquemontia mosaic Yucatan virus	JacMYuV	JQ821386
Vigna yellow mosaic virus	ViYMV	KC430936
Squash leaf curl virus	SLCV	M38183
Cabbage leaf curl virus	CbLCV	U65529
Rhynchosia golden mosaic Yucatán virus	RhGMYuV	EU021216
Tomato severe leaf curl virus	ToSLCV	JN680352
Jacquemontia yellow vein virus	JacYVV	KY624376
Tomato rugose yellow leaf curl virus	TRYLCV	JN381819
Tomato common mosaic virus	ToCmMV	EU710754
**Curtoviruses-type II** **QYKVSGGTKANKDDVYHN**		
Spinach severe surly top virus	SpSCTV	GU734126
Horseradish curly top virus	HrCTV	U49907
**Old and New World Begomoviruses** **FQIDGRSARGGQQTAND**		
Abutilon Brazil virus	AbBV	FN434438
Pepper huasteco yellow vein	PHYVV	NC_001359
Jacquemontia yellow mosaic virus	JacYMV	KF661331
Bean golden yellow mosaic virus	BGYMV	NC_038791
African cassava mosaic virus	ACMV	X17095
Watermelon chlorotic stunt virus	WmCSV	NC_003708
Indian cassava mosaic virus	ICMV	Z24758
Sweet potato leaf curl virus	SwLCV	MK931322
**Curtoviruses type-I** **FQIDGRSARGGQQTAND**		
Beet curly top virus - California [Logan]	BCTV-Cal	NC_001412
Beet curly top virus- Mx	BCTV-MX	EU586261
**Turncurtoviruses** **FQIDGRSARGGQQTAND**		
Turnip curly top virus	TCTV	KX533468
Turnip leaf roll virus	TLRoV,	KT388088
Sesame curly top virus	SesCTV	MH595454
**Topocovirus** **FQIDGRSARGGQQTAND**		
Tomato pseudo-curly top	TPCTV	X84735

Geminiviruses unassigned to a genus<br>FQIDGRSARGGQQTAND		
Grapevine geminivirus	GraGV-A	KX570610
Apple geminivirus	AGmV,	KM386645
Juncus maritimus associated virus	JmaV	MG001958
**Endogenous viral sequences (EVS)**<br>**FQIDGRSARGGQQTAND**		
Dioscorea cirrhosa endogenous viral seq.	EVS-*Dioscorea*	KJ629235
Nicotiana tomentosiformis geminivirus-like replication protein	EVS-*Nicotiana*	XM_009626235
Corchorus yellow vein virus-like DNA	EVS-*Corchorus*	KX10121
