# Supplementary material for: A Lineage of Begomoviruses Encode Rep and AC4 Proteins of Enigmatic Ancestry: Hints on the Evolution of Geminiviruses in the New World
Source: Viruses. 2019 Jul 13;11(7):644. doi: 10.3390/v11070644 (PMC6669703; doi:10.3390/v11070644)
Supplement: Supplementary file 1 [file viruses-11-00644-s001.zip › Suppl. Table S3- Torres-Herrera et al..pdf]

A lineage of begomoviruses encode Rep and AC4 proteins of enigmatic ancestry: hints on the evolution of geminiviruses in the New World.

Iliana Torres-Herrera[1,5*], Angélica Romero-Osorio[1*], Oscar Moreno-Valenzuela[2], Guillermo Pastor Palacios[3], Yair Cardenas-Conejo[4], Jorge H. Ramírez-Prado[2], Lina Riego-Ruiz[1], Yereni Minero-García[2], Salvador Ambriz-Granados[1], Gerardo R. Argüello-Astorga [1&].

[1] División de Biología Molecular, Instituto Potosino de Investigación Científica y Tecnológica, A.C., San Luís Potosí, SLP, México.

[2] Centro de Investigación Científica de Yucatán, A.C., Mérida, Yucatán, México

[3] CONACYT–CIIDZA–Instituto Potosino de Investigación Científica y Tecnológica A.C., San Luis Potosí, SLP, México,

[4] CONACyT-Universidad de Colima, Colima, México.

[5] Facultad de Ciencias Forestales, Universidad Juárez del Estado de Durango, México.

Supplementary Table S3. Names, acronyms and GenBank accession numbers of geminiviruses and endogenous viral *sequences (EVS)* included in the alignments of the N-terminal domain of AC4/C4 proteins (Figure 7).

Table S3 – Geminivirus and endogenous virus sequence in the alignment of N-terminal domain of AC4 proteins.

**SLCV clade begomoviruses**<br>**MKLFKCFK**		
Virus	Acronym	GenBank Accession
Abutilon golden mosaic Yucatan virus	AbGMYV	KC430935
Cabbage leaf curl virus	CbLCuV	MH248136
Bean calico mosaic virus	BCaMV	AF110189
Tomato common mosaic virus	ToCoMV	NC_010835
Sida yellow leaf curl virus	SiYLCV	EU710750
Tomato rugose yellow leaf curl virus	ToRYLCV	JN381815
**Curtoviruses-type II**<br>**MKLFKCFK**		
Horseradish curly top virus	HrCTV	U49907
Spinach severe curly top virus	SpSCTV	GU734126
**Old and New World Begomoviruses**<br>**MGNLIS**		
Tomato golden mosaic virus	TGMV	NC_001507
Rhynchosia golden mosaic virus	RhGMV	NC_010294
Tomato yellow leaf curl Vietnam virus	TYLCVV	EU189150
Mimosa yellow leaf curl virus	MiLCV	DQ641695
East African cassava mosaic Zanzibar virus	EACMZV	MK059410
Cassava mosaic Madagascar virus	CMMdV	HE617299
Curtoviruses- I<br>**MGNLIS**		
Beet curly top virus - California [Logan]	BCTV-Cal	NC_001412
Beet curly top virus-Idaho	BCTV-Idaho	KX867057
**Topocuviruses**<br>**MGNLIS**		
Tomato pseudo-curly top	TPCTV	X84735
**Unclassified geminiviruses**<br>**MGNLIS**		
Grapevine geminivirus A	GraGV-A	KX618694
Apple geminivirus	AGmV	KM386645
Juncus maritimus- associated virus	JmaV	MG001958
Polygala garcinii-associated virus	PgaV	MG001959

Endogenous viral sequences		
Lactuca sativa cultivar Salinas unplaced genomic scaffold	EVS-*Lactuca sativa*	NW_019676544
Corchorus olitorius cultivar JRO-524	EVS-*Corchorus olitorius*	LLWS00000000
Nicotiana tomentosiformis unplaced genomic scaffold	EVS-*Nicotiana tomentosiformis*	NW_008941319
Dioscorea nummularia endogenous virus EGV2	EVS- *Dioscorea nummularia*	KJ629225
Coffea arabica geminivirus-like replication protein	EVS-C*offea canephora*	XM_027241759
