# Supplementary material for: A Lineage of Begomoviruses Encode Rep and AC4 Proteins of Enigmatic Ancestry: Hints on the Evolution of Geminiviruses in the New World
Source: Viruses. 2019 Jul 13;11(7):644. doi: 10.3390/v11070644 (PMC6669703; doi:10.3390/v11070644)
Supplement: Supplementary file 1 [file viruses-11-00644-s001.zip › Supplementary Fig. 1 Torres Herrera et al..pdf]

A lineage of begomoviruses encode Rep and AC4 proteins of enigmatic ancestry: hints on the evolution of geminiviruses in the New World.

Iliana Torres-Herrera[1*], Angélica Romero-Osorio[1*], Oscar Moreno-Valenzuela[2], Guillermo Pastor Palacios[3], Yair Cardenas-Conejo[4], Jorge H. Ramírez-Prado[2], Lina Riego-Ruiz[1], Yereni Minero-García[2,] Salvador Ambriz-Granados[1], Gerardo R. Argüello-Astorga[1&].

Supplementary Figure 1.

Weeds infected with the begomoviruses described in this study

A

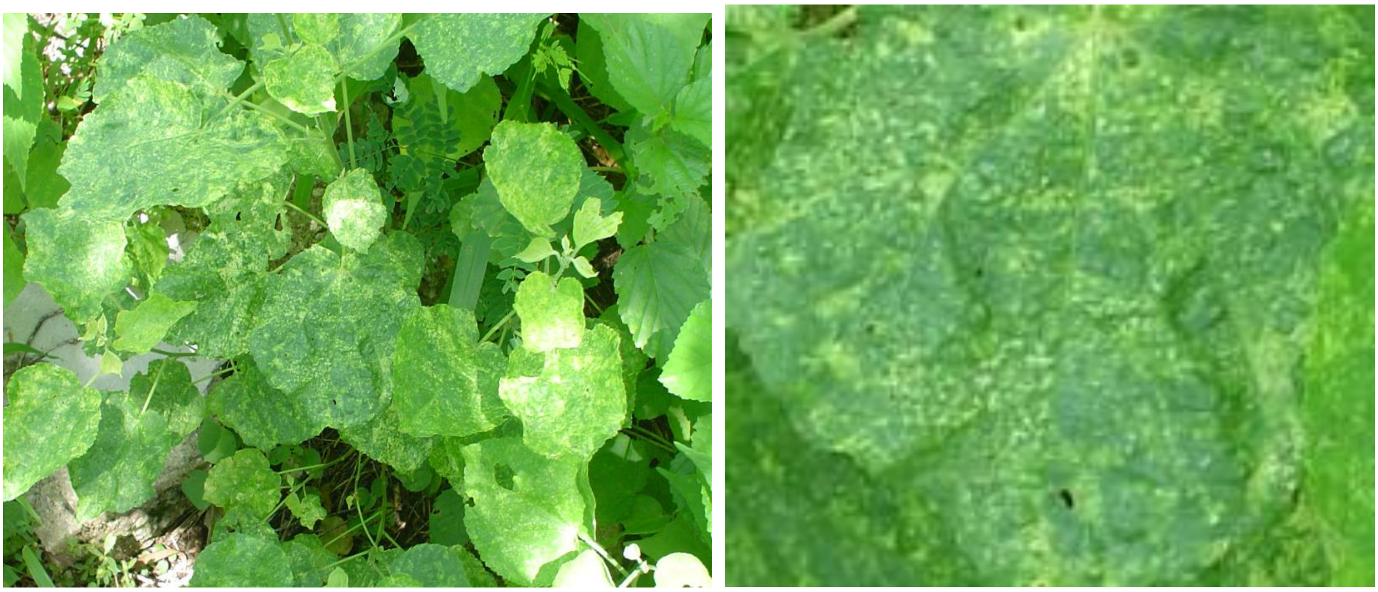

S1A. *Abutilon permolle* plant co-infected with AbGMYV and CoYSV.

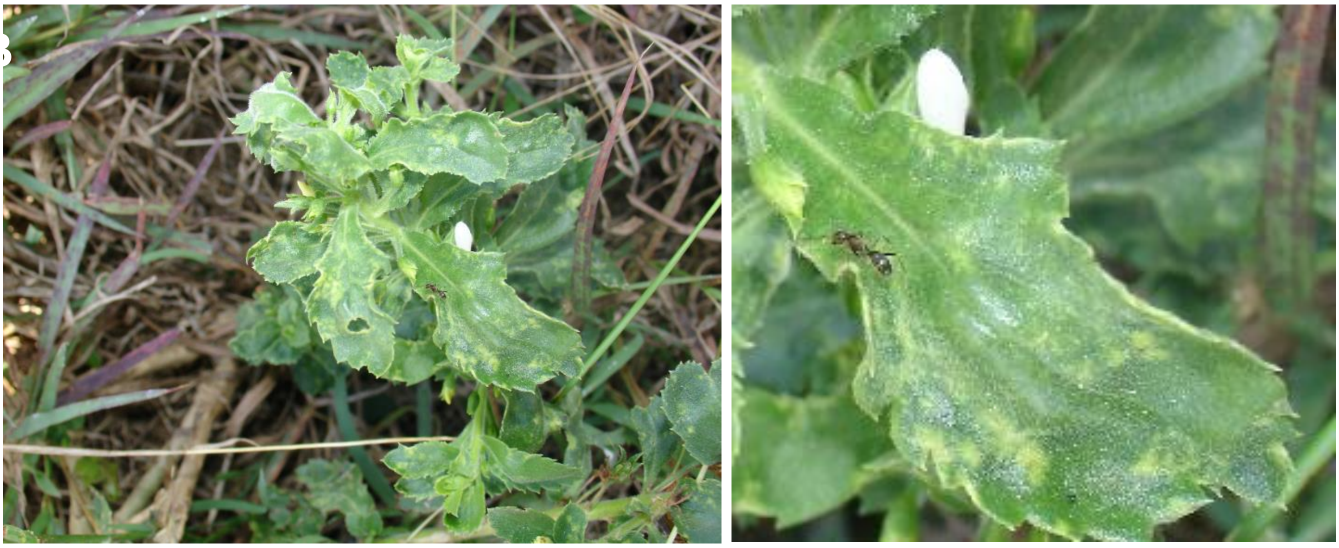

S1B. ***Capraria biflora*** **plant infected with CarYMYV**

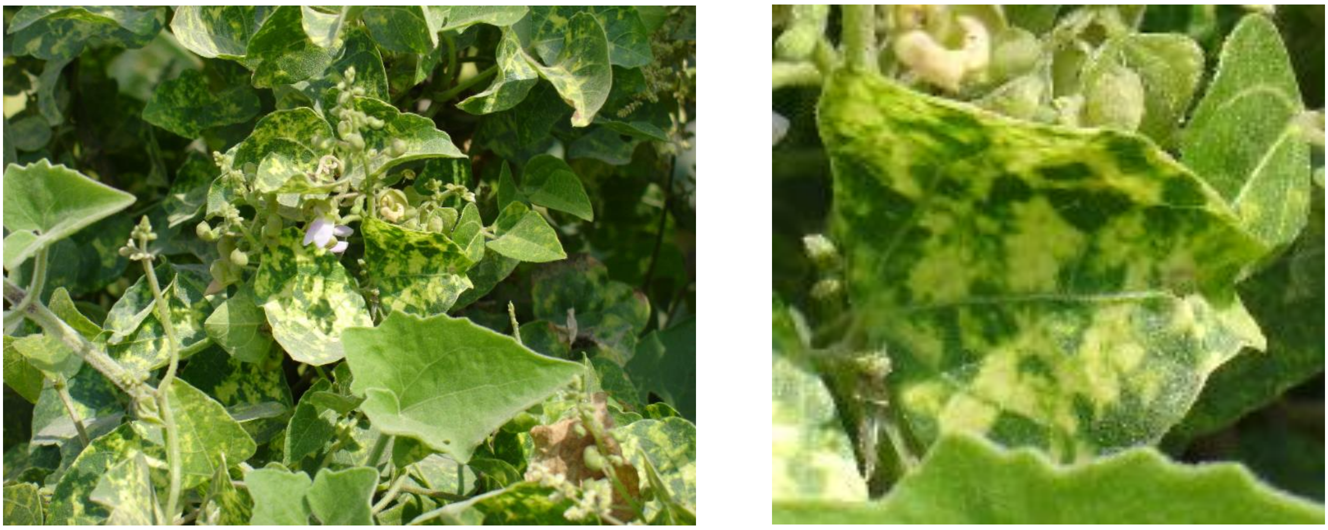

S1C. ***Vigna elegans*** **plant infected with ViYMV.**
