# Supplementary material for: A Lineage of Begomoviruses Encode Rep and AC4 Proteins of Enigmatic Ancestry: Hints on the Evolution of Geminiviruses in the New World
Source: Viruses. 2019 Jul 13;11(7):644. doi: 10.3390/v11070644 (PMC6669703; doi:10.3390/v11070644)
Supplement: Supplementary file 1 [file viruses-11-00644-s001.zip › Supplementary Figure S2 Torres-Herrera et al.pdf]

A lineage of begomoviruses encode Rep and AC4 proteins of enigmatic ancestry: hints on the evolution of geminiviruses in the New World.

Iliana Torres-Herrera[1,5*], Angélica Romero-Osorio[1*], Oscar Moreno-Valenzuela[2], Guillermo Pastor Palacios[3], Yair Cardenas-Conejo[4], Jorge H. Ramírez-Prado[2], Lina Riego-Ruiz[1], Yereni Minero-García[2], Salvador Ambriz-Granados[1], Gerardo R. Argüello-Astorga [1&].

[1] División de Biología Molecular, Instituto Potosino de Investigación Científica y Tecnológica, A.C., San Luís Potosí, SLP, México.

[2] Centro de Investigación Científica de Yucatán, A.C., Mérida, Yucatán, México

[3] CONACYT–CIIDZA–Instituto Potosino de Investigación Científica y Tecnológica A.C., San Luis Potosí, SLP, México,

[4] CONACyT-Universidad de Colima, Colima, Mexico.

[5] Facultad de Ciencias Forestales, Universidad Juárez del Estado de Durango, Mexico.

Supplementary Figure S2

Phylogenetic tree and matrix of pairwise sequence identity based on DNA-A sequences of the isolated begomoviruses and their relatives.

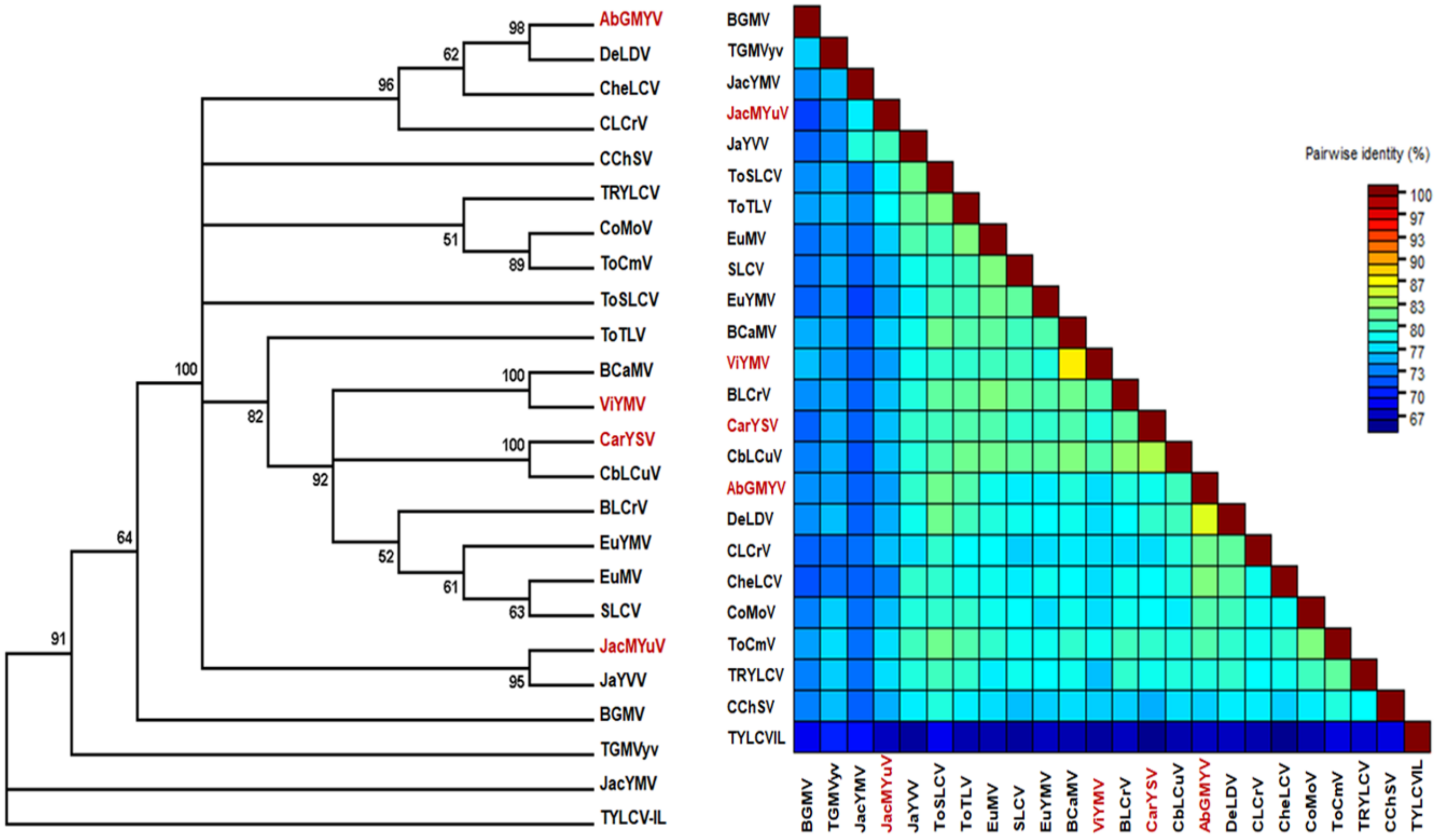

Supplementary Figure 2. Maximum likelihood phylogenetic tree and matrix of pairwise sequence identity based on full length sequences of genome components A. ML phylogenies were constructed by multiple alignments of complete DNA-A sequences. ML tree in MEGA 7 was based on the Tamura-Nei model and the tree support was tested by bootstrapping with 1000 replicates. SDTv software was used for the matrix of pairwise sequence identity.
