# Supplementary material for: A Lineage of Begomoviruses Encode Rep and AC4 Proteins of Enigmatic Ancestry: Hints on the Evolution of Geminiviruses in the New World
Source: Viruses. 2019 Jul 13;11(7):644. doi: 10.3390/v11070644 (PMC6669703; doi:10.3390/v11070644)
Supplement: Supplementary file 1 [file viruses-11-00644-s001.zip › Supplementary Figure S3- Torres-Herrera et al. 2019.pdf]

A lineage of begomoviruses encode Rep and AC4 proteins of enigmatic ancestry: hints on the evolution of geminiviruses in the New World.

Iliana Torres-Herrera[1,5*], Angélica Romero-Osorio[1*], Oscar Moreno-Valenzuela[2], Guillermo Pastor Palacios[3], Yair Cardenas-Conejo[4], Jorge H. Ramírez-Prado[2], Lina Riego-Ruiz[1], Yereni Minero-García[2], Salvador Ambriz-Granados[1], Gerardo R. Argüello-Astorga [1&].

[1] División de Biología Molecular, Instituto Potosino de Investigación Científica y Tecnológica, A.C., San Luís Potosí, SLP, México.
[2] Centro de Investigación Científica de Yucatán, A.C., Mérida, Yucatán, México
[3] CONACYT–CIIDZA–Instituto Potosino de Investigación Científica y Tecnológica A.C., San Luis Potosí, SLP, México,
[4] CONACyT-Universidad de Colima, Colima, Mexico.
[5] Facultad de Ciencias Forestales, Universidad Juárez del Estado de Durango, Mexico.

Supplementary Figure 3.

Recombination event in the AbGMYuV DNA-A detected by RDP

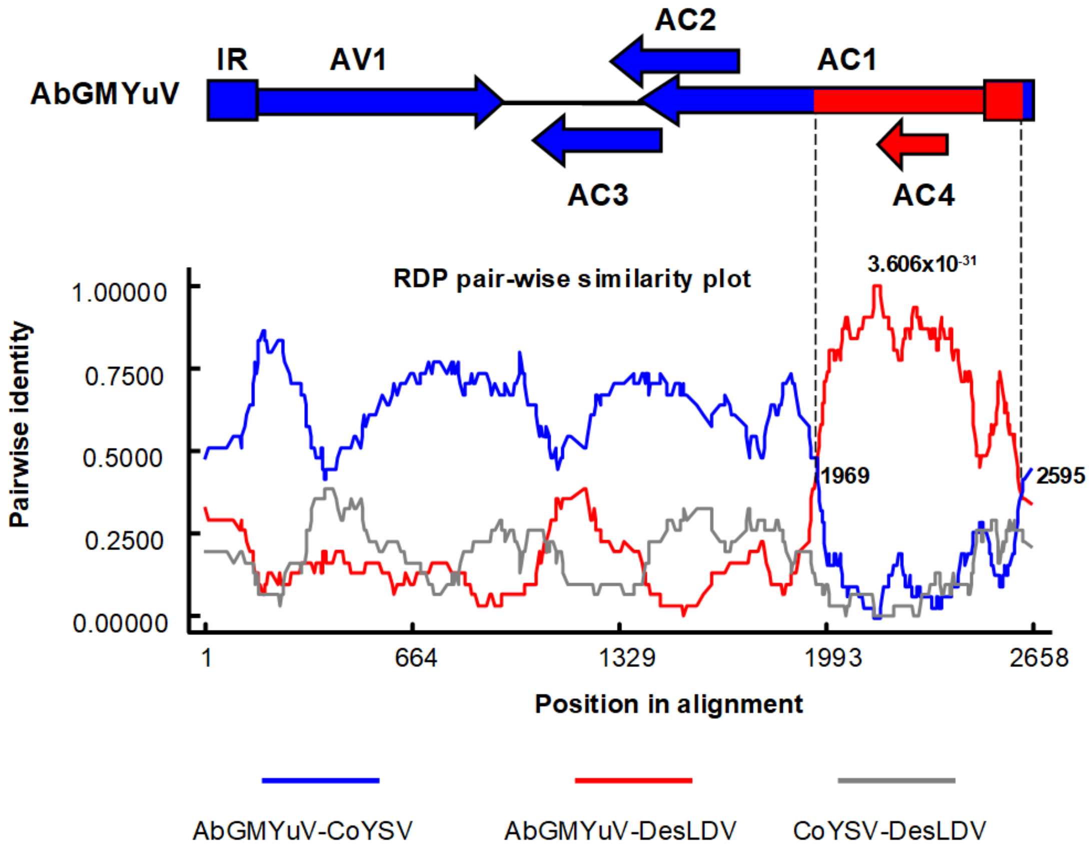

Supplementary Figure 3. Recombination event in the AbGMYuV DNA-A detected by RDP 4. The schematic representation of AbGMYuV genomic component A is at the top of the figure and the RDP pair-wise similarity plot is shown below. The blue region indicates the major parent section (CoYSV) and the red region the minor parent (DeLDV). The color lines in the similarity plot indicate the comparison between two virus sequences. The beginning breakpoint (1969 nt) and the ending breakpoint (2595 nt) are indicated by the vertical lines.
