# Supplementary material for: A Lineage of Begomoviruses Encode Rep and AC4 Proteins of Enigmatic Ancestry: Hints on the Evolution of Geminiviruses in the New World
Source: Viruses. 2019 Jul 13;11(7):644. doi: 10.3390/v11070644 (PMC6669703; doi:10.3390/v11070644)
Supplement: Supplementary file 1 [file viruses-11-00644-s001.zip › Supplementary Figure S4- Torres-Herrera et al..pdf]

A lineage of begomoviruses encode Rep and AC4 proteins of enigmatic ancestry: hints on the evolution of geminiviruses in the New World.

Iliana Torres-Herrera[1,5*], Angélica Romero-Osorio[1*], Oscar Moreno-Valenzuela[2], Guillermo Pastor Palacios[3], Yair Cardenas-Conejo[4], Jorge H. Ramírez-Prado[2], Lina Riego-Ruiz[1], Yereni Minero-García[2], Salvador Ambriz-Granados[1], Gerardo R. Argüello-Astorga [1&].

[1] División de Biología Molecular, Instituto Potosino de Investigación Científica y Tecnológica, A.C., San Luís Potosí, SLP, México.
[2] Centro de Investigación Científica de Yucatán, A.C., Mérida, Yucatán, México
[3] CONACYT–CIIDZA–Instituto Potosino de Investigación Científica y Tecnológica A.C., San Luis Potosí, SLP, México,
[4] CONACyT-Universidad de Colima, Colima, Mexico.
[5] Facultad de Ciencias Forestales, Universidad Juárez del Estado de Durango, Mexico.

Supplementary Figure S4

Phylogenetic relationships between the novel begomoviruses and selected New World and Old World begomoviruses

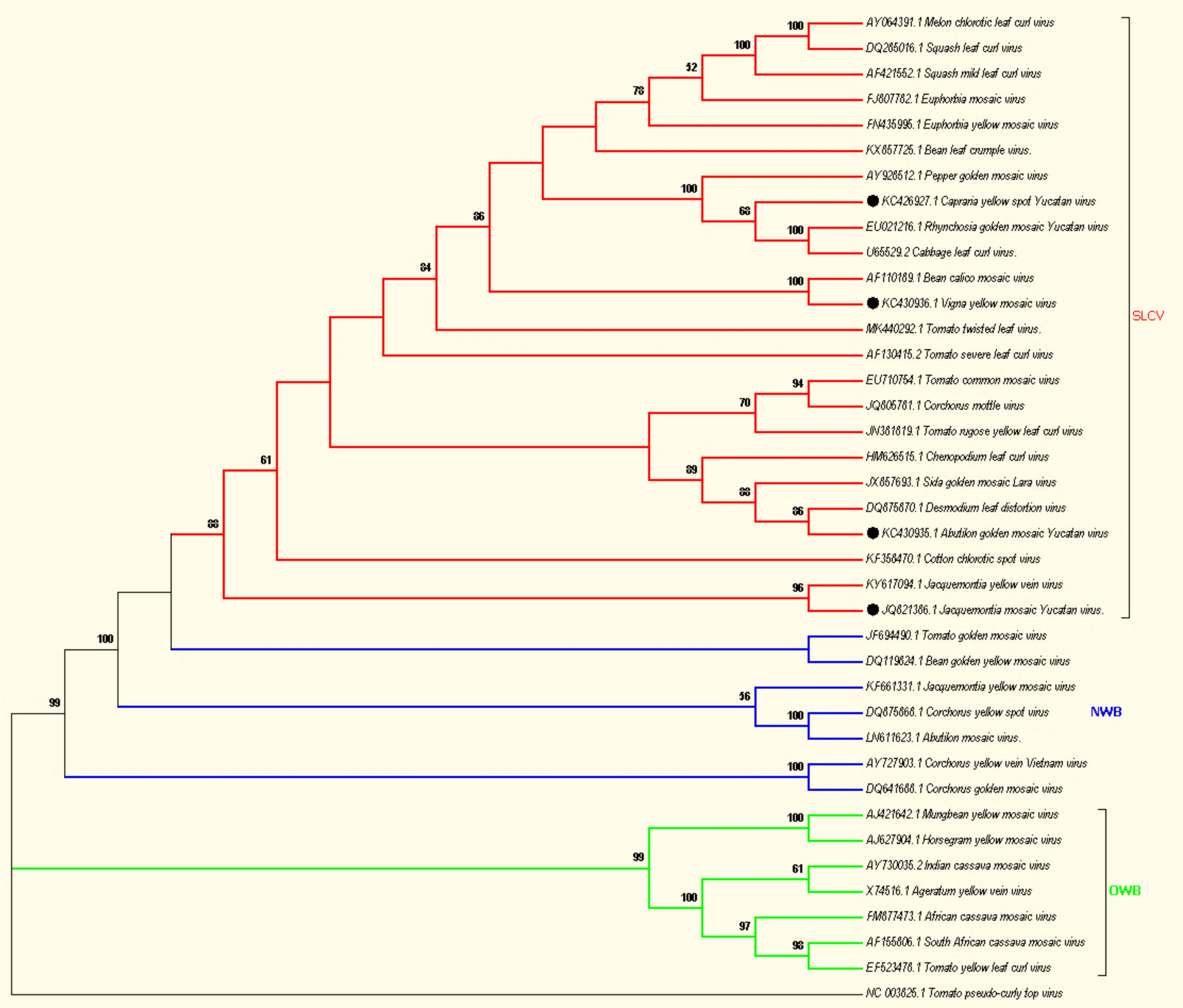

Supplementary Figure 4. Phylogenetic relationships between the novel begomoviruses (highlighted with black dots) and selected NW and OW begomoviruses. A multiple sequence alignment was constructed using the MAFFT algorithm v.7.017 implemented on Geneious v.6.1.8 using the default parameters. The evolutionary history was inferred by using the Maximum Likelihood method and General Time Reversible model GTR [Nei and Kumar, 2000]. The tree with the highest log likelihood (-63810.98) is shown. The percentage of trees in which the associated taxa clustered together is shown next to the branches (1000 bootstrap replicates). The topocuvirus *Tomato pseudo-curly top virus* was used as outgroup.
