# Supplementary material for: A Lineage of Begomoviruses Encode Rep and AC4 Proteins of Enigmatic Ancestry: Hints on the Evolution of Geminiviruses in the New World
Source: Viruses. 2019 Jul 13;11(7):644. doi: 10.3390/v11070644 (PMC6669703; doi:10.3390/v11070644)
Supplement: Supplementary file 1 [file viruses-11-00644-s001.zip › Supplementary Figure S5- Torres-Herrera et al.pdf]

A lineage of begomoviruses encode Rep and AC4 proteins of enigmatic ancestry: hints on the evolution of geminiviruses in the New World.

Iliana Torres-Herrera[1,5*], Angélica Romero-Osorio[1*], Oscar Moreno-Valenzuela[2], Guillermo Pastor Palacios[3], Yair Cardenas-Conejo[4], Jorge H. Ramírez-Prado[2], Lina Riego-Ruiz[1], Yereni Minero-García[2], Salvador Ambriz-Granados[1], Gerardo R. Argüello-Astorga [1&].

[1] División de Biología Molecular, Instituto Potosino de Investigación Científica y Tecnológica, A.C., San Luís Potosí, SLP, México.
[2] Centro de Investigación Científica de Yucatán, A.C., Mérida, Yucatán, México
[3] CONACYT–CIIDZA–Instituto Potosino de Investigación Científica y Tecnológica A.C., San Luis Potosí, SLP, México,
[4] CONACyT-Universidad de Colima, Colima, Mexico.
[5] Facultad de Ciencias Forestales, Universidad Juárez del Estado de Durango, Mexico.

Supplementary Figure S5

DNA-B inferred evolutionary relationships of viruses isolated in this work compared with selected begomoviruses

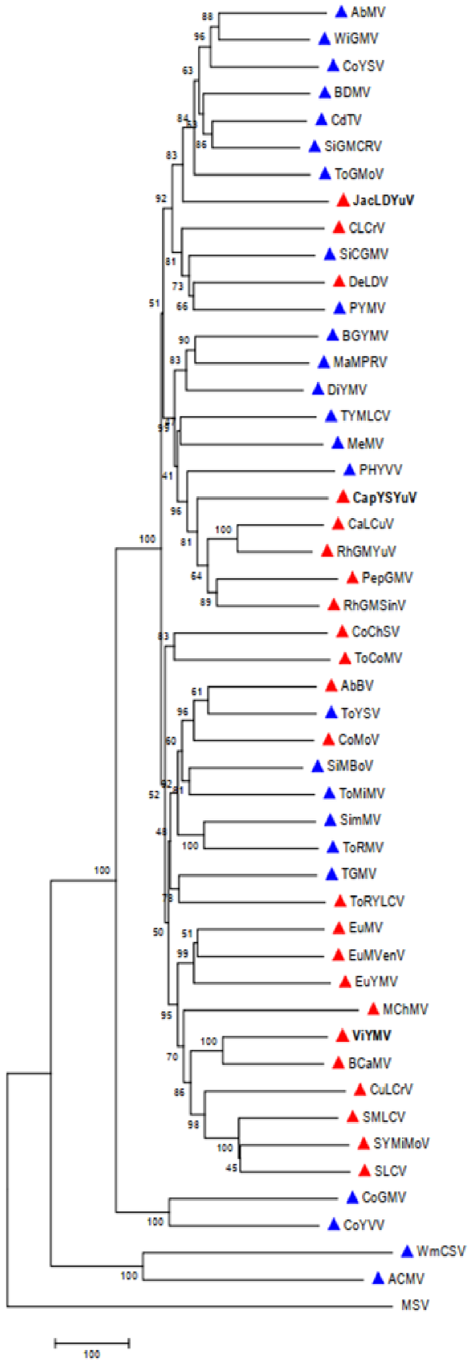

Supplementary Figure 5. DNA-B inferred evolutionary relationships of viruses isolated in this work compared with selected begomoviruses. Phylogenetic tree indicating the relationship between the DNA-B genomic component of viruses isolated in this work (AbGMYuV, CarYSYuV, JacMYuV and, ViYMV) and selected begomoviruses is shown. The SLCV clade members are denoted with a red triangle depicted before the name. *Maize streak virus* (MSV, a mastrevirus) was used as outgroup. Genome B phylogeny was inferred with a total of 1470 positions in the final dataset using the Neighbor-Joining method. The tree is drawn to scale, with branch lengths in the same units as those of the evolutionary distances used to infer the phylogenetic tree. The evolutionary distances were computed using the number of differences method and are in the units of the number of base differences per sequence. The analysis involved 46 nucleotide sequences. All positions containing gaps and missing data were eliminated. Tree support was tested by bootstrapping with 1000 replicates.
