# Supplementary material for: A Lineage of Begomoviruses Encode Rep and AC4 Proteins of Enigmatic Ancestry: Hints on the Evolution of Geminiviruses in the New World
Source: Viruses. 2019 Jul 13;11(7):644. doi: 10.3390/v11070644 (PMC6669703; doi:10.3390/v11070644)
Supplement: Supplementary file 1 [file viruses-11-00644-s001.zip › Supplementary Figure S6-Torres-Herrera et al..pdf]

A lineage of begomoviruses encode Rep and AC4 proteins of enigmatic ancestry: hints on the evolution of geminiviruses in the New World.

Iliana Torres-Herrera[1,5*], Angélica Romero-Osorio[1*], Oscar Moreno-Valenzuela[2], Guillermo Pastor Palacios[3], Yair Cardenas-Conejo[4], Jorge H. Ramírez-Prado[2], Lina Riego-Ruiz[1], Yereni Minero-García[2], Salvador Ambriz-Granados[1], Gerardo R. Argüello-Astorga [1&].

[1] División de Biología Molecular, Instituto Potosino de Investigación Científica y Tecnológica, A.C., San Luís Potosí, SLP, México.
[2] Centro de Investigación Científica de Yucatán, A.C., Mérida, Yucatán, México
[3] CONACYT–CIIDZA–Instituto Potosino de Investigación Científica y Tecnológica A.C., San Luis Potosí, SLP, México,
[4] CONACyT-Universidad de Colima, Colima, Mexico.
[5] Facultad de Ciencias Forestales, Universidad Juárez del Estado de Durango, Mexico.

Supplementary Figure 6

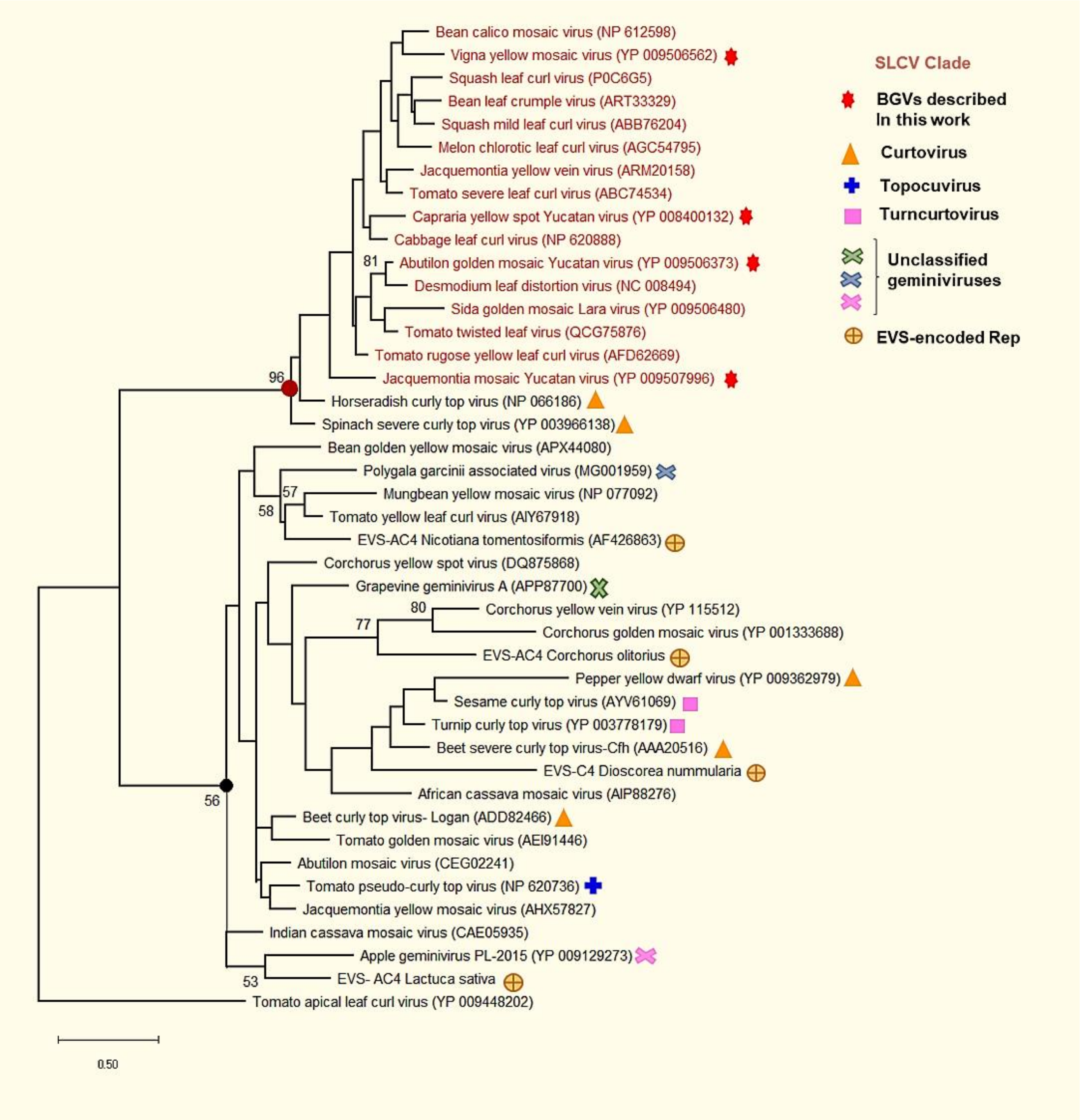

Supplementary Figure 6. Phylogenetic relationships between AC4 proteins encoded by SLCV-Lin members (highlighted in red) and those of other geminivirus subgroups. The phylogenetic tree was inferred by using the Maximum Likelihood method based on the JTT matrix-based model. The percentage of trees in which the associated taxa clustered together is shown next to the branches (1000 iterations). Red filled circle indicates the node of the S-Lin BGVs, and the black filled circle indicates the main branch of other geminiviruses.
